# Aquaporin-4 cerebrospinal fluid levels are higher in neurodegenerative dementia: looking at glymphatic system dysregulation

**DOI:** 10.1186/s13195-022-01077-6

**Published:** 2022-09-17

**Authors:** Andrea Arighi, Marina Arcaro, Giorgio Giulio Fumagalli, Tiziana Carandini, Anna Margherita Pietroboni, Luca Sacchi, Chiara Fenoglio, Maria Serpente, Federica Sorrentino, Giovanni Isgrò, Federico Turkheimer, Elio Scarpini, Daniela Galimberti

**Affiliations:** 1 Fondazione IRCCS Ca’ Granda Ospedale Maggiore Policlinico, Neurodegenerative Disease Unit, via Francesco Sforza 35, 20122 Milan, Italy; 2 University of Milan, Milan, Italy; 3 Department of Neuroimaging, Institute of Psychiatry, Psychology and Neuroscience, King’s College London, London, UK

**Keywords:** Glymphatic system, Aquaporin 4, Amyloid-β, Cerebrospinal fluid, Alzheimer’s disease, Frontotemporal dementia

## Abstract

Aquaporin-4 (AQP4) is a channel protein that plays a fundamental role in glymphatic system, a newly described pathway for fluid exchange in the central nervous system, as well as a central figure in a fascinating new theory for the pathophysiology of neurodegenerative diseases such as Alzheimer’s disease (AD) and frontotemporal dementia (FTD). In this study, cerebrospinal fluid (CSF) concentration of AQP4, amyloid-β, total tau and P-tau were determined in 103 CSF samples from patients affected by neurodegenerative dementias (AD and FTD) or psychiatric diseases and 21 controls. Significantly higher levels of AQP4 were found in AD and FTD patients compared to subjects not affected by neurodegenerative diseases, and a significant, positive correlation between AQP4 and total tau levels was found. This evidence may pave the way for future studies focused on the role of this channel protein in the clinical assessment of the glymphatic function and degree of neurodegeneration.

## Introduction

Interstitial fluid and solute clearance are crucial for homeostasis. Although in peripheral tissues this function is carried out by the well-characterised lymphatic system, the central nervous system (CNS)—which is one of the organs with the highest metabolic rate [1]—was thought to be devoid of such circulatory system, and these essential roles were considered prerogative of intra- and extra-cellular degradation mechanisms, such as autophagy and ubiquitination [2]. Furthermore, the removal of some critical proteins is indispensable for the health of the CNS, as their accumulation is a well-known pathogenetic mechanism for neurodegenerative diseases such as Alzheimer’s disease (AD) [3]. Recent studies have undoubtedly proven that the cerebrospinal fluid (CSF) continuously interchanges with interstitial fluid (ISF), thanks to a convective bulk flow movement facilitated by water channels, called aquaporin-4 (AQP4) [4, 5], which are highly polarised and expressed selectively on the astrocytic endfeet that surround the brain vasculature, both in the periarteriolar and the perivenular space [6]. The continuous CSF and ISF movement that is generated has been therefore named glymphatic system, because of its resemblance with the peripheral lymphatic system and the importance of the astroglia in its composition [4]. One of the main functions of the glymphatic system is, as a matter of fact, the clearance of solutes, and amyloid-β (Aβ) was demonstrated to follow this pathway for its elimination [4, 7]. Moreover, in AD, glymphatic activity is strongly compromised and this may contribute to the accumulation of Aβ [7–9]. Further studies demonstrated that patients suffering from neurodegenerative diseases show pathogenetic changes on a molecular level of the glymphatic system, which include a reactive gliosis—or neuroinflammation—of astrocytes, and loss of polarisation in the expression of AQP4 on the plasma membrane [6, 10, 11]. Whether it is a compromised glymphatic that causes protein accumulation or the amyloid plaques and neurofibrillary tangles that reduce the glymphatic flow and generate these changes is not yet completely known, but the main theory is that the two factors are in a mutual relationship, so that a feed-forward mechanism takes place [7].

Since AQP4 plays a key role in both the physiological and the pathological glymphatic system, one of the most interesting and appealing approaches, as well as the purpose of this study, is to investigate the changes in AQP4 concentration in the CSF, obtained by lumbar puncture, that is routinely performed when a neurodegenerative disease is suspected.

## Materials and methods

### Subjects

A total of 124 subjects were recruited for this study, 103 of whom were patients undergoing neurological workup at the Neurodegenerative Diseases Unit of the Fondazione IRCCS Ca’ Granda Ospedale Maggiore Policlinico in Milan (Italy), between January 2012 and February 2020, whereas 21 were healthy controls. The clinical workup included past medical history; general and neurological examination; neurocognitive assessment—including neuropsychological tests such as Mini-Mental State Examination (MMSE); magnetic resonance imaging; and lumbar puncture. Control subjects underwent lumbar puncture in suspicion of pathologies of the CNS and were discharged with no evidence of CNS involvement. Therefore, we considered them as controls.

Subjects were assessed at baseline and during the follow-up and diagnoses were established according to multiparametric criteria: the International Working Group 2 (IWG-2) Criteria for Alzheimer’s Disease Diagnosis [12] for diagnosis of AD, the revised diagnostic criteria for the behavioural variant of frontotemporal dementia by Rascovsky et al. [13] for the diagnosis of FTD, and Petersen et al. criteria [14] were considered to diagnose MCI patients, who were assessed periodically and maintained their cognitive functions for 4 years. Psychiatric disorders (which were major depressive disorder and bipolar disorder) were evaluated according to the Diagnostic and Statistical Manual of Mental Disorders Fifth Edition (DSM-5) [15].

### CSF collection and protein determination

Lumbar puncture was performed in the L3/L4 or L4/L5 interspace between 8 and 10 am after a night of fasting. The samples were later centrifuged at 1500 × g for 10 min at 4 °C. The supernatants were aliquoted in polypropylene tubes and stored at − 80 °C until use. Before freezing them, the samples were analysed and CSF cell count, glucose, and total protein were calculated. CSF levels of Aβ1–42, Aβ1–40, tau, and P-tau of 101 subjects were assessed using the method ChemiLuminescence Enzyme ImmunoAssay (CLEIA) by a Lumipulse G600II platform (Fujirebio).

These evaluations allowed to stratify patients according to their pathophysiological background basing on the amyloid/tau/neurodegeneration (ATN) classification of the National Institute on Aging – Alzheimer's Association (NIA-AA) Research Framework. This classification is based on dichotomous categories (normal/abnormal) of Aβ (A), tau (T), and neurodegeneration (N) biomarkers [16]. CSF concentration threshold for Aβ1–42 was 640 pg/mL (subjects with CSF concentrations greater than this value were considered A − , normal; if it was lower they were considered A + , abnormal, with Aβ deposition); the threshold for tau protein was set at 580 pg/mL (if they had concentrations greater than this value, they were considered N + , abnormal, with tau deposition; otherwise they were N − , normal); the threshold for P-Tau was 61 pg/mL (if it was greater, patients were labelled T + , abnormal, with neurodegeneration; if not, T − , normal) [17].

AQP4 concentration in the CSF was determined with immunoassay from Cusabio for the whole population considered for the study; quantitative enzyme immunoassay technique includes an AQP4 antibody pre-coated into a microplate, about 100 μl of standards and samples are pipetted into the wells and each AQP4 present is bound by the immobilised antibody. After removing any unbound substances, a biotin-conjugated antibody specific for AQP4 is added and then, after washing, avidin conjugated Horseradish Peroxidase (HRP) is also added followed by the substrate solution and the colour developed is in proportion to the amount of AQP4 bound in the initial step. The optical density was determined using a micro reader set to 450 nm. The sensitivity of this assay or Lower Limit of Detection of human AQP-4 is typically less than 39 pg/ml as reported by the manufacturer (www.cusabio.com).

### Statistical analyses

Data were analysed using the statistical spreadsheets Jamovi v 1.8.1 (https://www.jamovi.org) and JASP v 0.16.3 (https://jasp-stats.org/). AQP4 and other CSF biomarker levels, demographic parameters such as age and disease at the time of the lumbar puncture, and educational stage as well as MMSE score were all considered continuous variables and expressed as mean ± standard error of the mean (SEM). Conversely, patients’ sex, diagnostic group, and the dichotomised classification on AQP4 levels compared to the median value were all considered categorical variables. Significant statistical threshold was set at 0.05. Shapiro–Wilk’s test of normality was performed for continuous variables, and intergroup comparisons were carried out using the non-parametric Kruskal–Wallis test and Mann–Whitney test. Spearman’s correlation was used to assess the association between CSF biomarkers. Multivariate linear regressions were used to explore the relation between different CSF biomarkers, and demographics were used as covariates. Categorical variables were analysed using the chi-squared test. With respect to the patients’ progression in MMSE scores for at least 4 years of follow-up, Kaplan–Meier estimators were realised, considering the conversion to dementia (from a MMSE score ≥ 24 to a MMSE score < 24) as the main event.

## Results

Fifty-nine females and 65 males were included in the study. Patients were divided into groups, depending on their diagnosis: 48 of these subjects were affected by AD, 22 were suffering from FTD, 20 had mild cognitive impairment with no evidence of a neurodegenerative underlying pathology, 13 had psychiatric disorders, and 21 belonged to the control group. Subjects were later grouped again into three main groups, which were patients suffering from neurodegenerative disease (AD and FTD), patients with cognitive deficits but with no diagnosis of neurodegenerative disease (MCI and psychiatric patients), and controls. Finally, only two groups were analysed and compared to each other: one was composed of patients with a diagnosis of neurodegenerative disease, whereas the other was obtained by gathering all the other subjects. Furthermore, the NIA-AA ATN classification was considered, and 101 of the 124 patients—the ones whose concentrations of specific proteins measured using CLEIA were available—were divided into groups according to A, T, and N parameters (depending on their CSF levels of, respectively, Aβ, Tau, and P-Tau).

Principal sociodemographic and clinical characteristics of studied subjects are summarised in Table 1. There were no significant differences between groups with respect to sex, educational stage, or disease duration. Conversely, as expected, there were statistically significant differences between age in the AD patients compared to the psychiatric group (*p* = 0.034) and in MMSE score between AD patients and the not neurodegenerative groups (Kruskal–Wallis test: AD versus controls *p* < 0.001, AD versus MCI *p* < 0.001, AD versus psychiatric patients *p* < 0.001), between subjects affected by FTD and controls (Kruskal–Wallis test: *p* = 0.002), as well as between MCI groups and controls (*p* = 0.035). These differences maintained their significance when the main groups were considered (degenerative patients versus not degenerative Mann–Whitney test: for age *p* = 0.015; for MMSE *p* < 0.001).

**Table 1:** Principal sociodemographic and clinical characteristics of studied subjects: Alzheimer’s disease (AD), behavioural variant frontotemporal dementia (bvFTD), mild cognitive impairment (MCI), psychiatric disorders (Psy), and controls (Con). Values are expressed as mean ± SEM

	AD	bvFTD	MCI	Psy	Con
Number	48	22	20	13	21
Age (years)	69.8 ± 1.1	70.5 ± 1.5	67.3 ± 1.9	62.8 ± 2.3	70.1 ± 1.1
Gender (M:F)	22:26	10:12	10:10	8:5	15:6
Education (years ± SEM)	10.0 ± 0.6	9.9 ± 1.0	10.1 ± 0.8	13.0 ± 1.2	10.4 ± 0.9
Disease duration (years ± SEM)	3.1 ± 0.3	2.3 ± 0.3	2.7 ± 0.4	5.7 ± 2.7	4.0 ± 0.9
MMSE ± SEM	21.2 ± 0.9	23.1 ± 1.2	25.9 ± 0.5	27.2 ± 0.7	27.4 ± 1.0
Beta-amyloid (pg/ml ± SEM)	397.6 ± 20.9	586.3 ± 47.6	760.7 ± 69.6	844.9 ± 124.2	805.2 ± 72.8
Total tau (pg/ml ± SEM)	909.8 ± 72.8	552.2 ± 83.7	306.8 ± 29.1	253.0 ± 31.8	312.1 ± 25.7
P-Tau (pg/ml ± SEM)	149.6 ± 11.3	76.2 ± 13.8	42.3 ± 4.4	36.3 ± 5.8	48.3 ± 5.0
AQP4 (pg/ml ± SEM)	350.4 ± 32.7	310.5 ± 34.6	266.2 ± 25.0	261.6 ± 32.1	209.6 ± 23.4

*MMSE* Mini-Mental State Examination, *AQP4* Aquaporin 4

AQP4 levels were not normally distributed (Shapiro–Wilk *p* < 0.001) with a mean of 296.6 ± 16.0 pg/ml. There was no correlation between AQP4 concentration and demographic parameters (Spearman’s correlation: educational stage *p* = 0.268; age at lumbar puncture = 0.413; disease duration *p* = 0.390; Mann–Whitney test: sex *p* = 0.453) except for MMSE score at the time lumbar puncture was performed (Spearman’s correlation: rho =  − 0.160 *p* = 0.038).

**Fig. 1:**
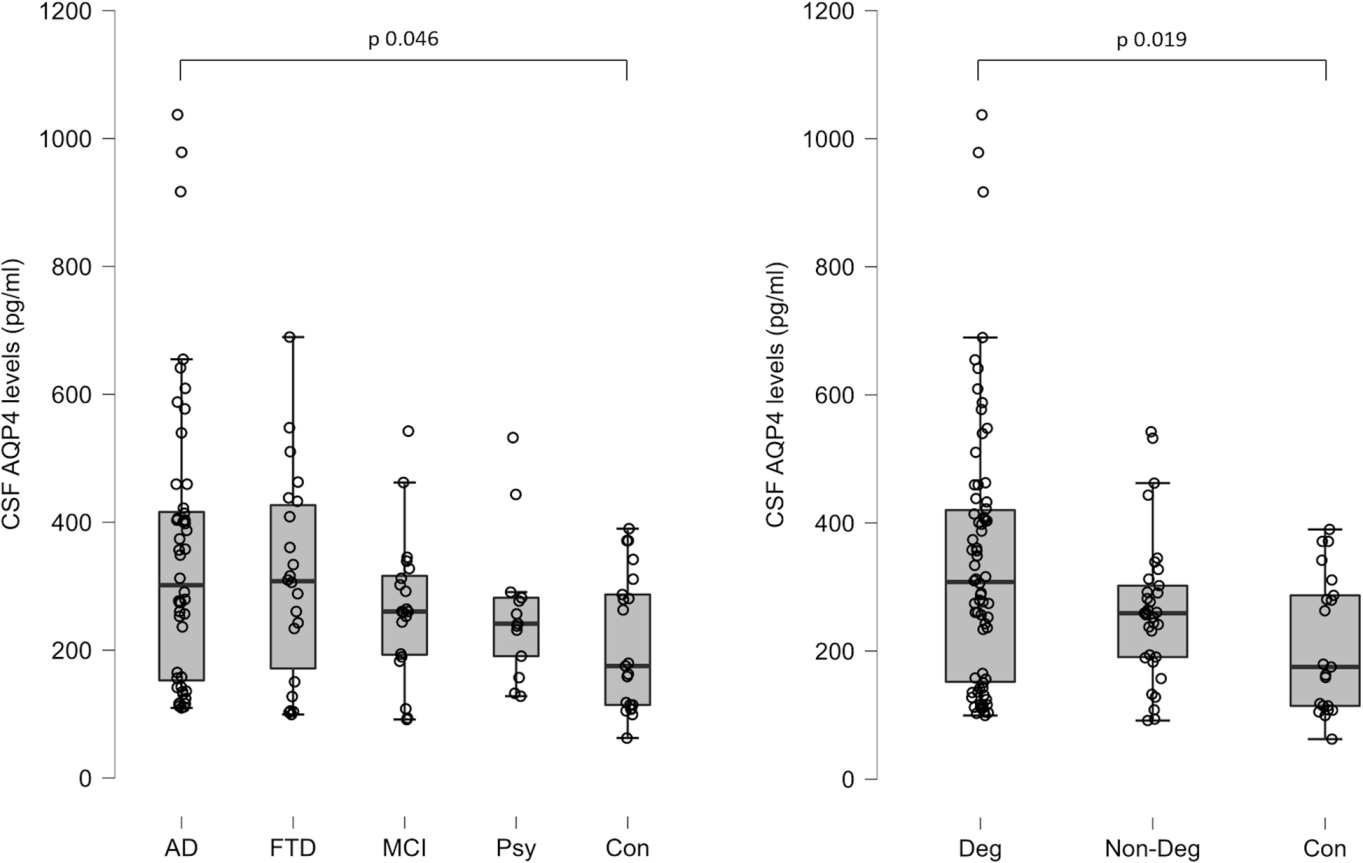
On the left, box plot representing cerebrospinal fluid (CSF) aquaporin 4 (AQP4) levels in the diagnostic groups: Alzheimer’s disease (AD), frontotemporal dementia (FTD), mild cognitive impairment (MCI), psychiatric disorder (Psy), and control group (Con). On the right, box plot with three-group classification: patients affected by neurodegenerative disease (Deg), patients with neurological or psychiatric not degenerative disease (Non-Deg), and controls (Con)

When the single diagnostic groups were taken into consideration, there was a significant difference in AQP4 levels between AD patients and the control group (AD 350.4 ± 32.7 pg/ml vs controls 209.6 ± 23.4 pg/ml, Dwass-Steel-Critchlow-Fligner test for pairwise comparisons from Kruskal–Wallis test: *p* = 0.046), but no other statistically significant variations were obtained (FTD vs controls *p* = 0.347, MCI vs controls *p* = 0.713, psychiatric patients vs controls *p* = 0.759) (Fig. 1).

**Fig. 2:**
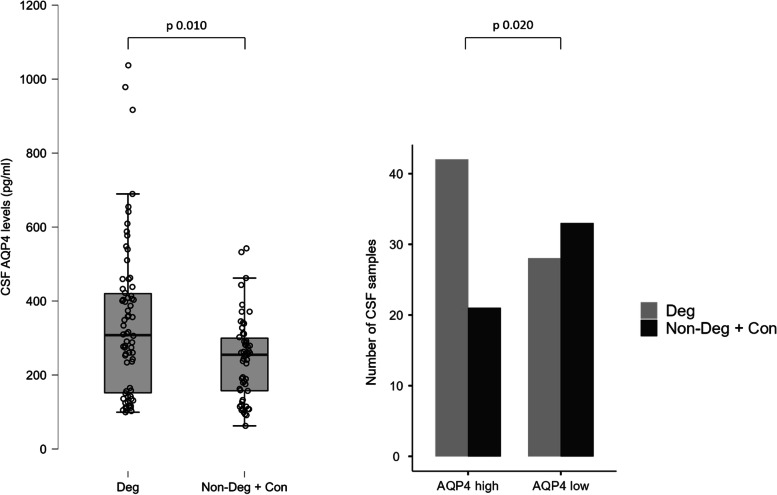
Box plot representing cerebrospinal fluid (CSF) aquaporin 4 (AQP4) levels (on the left) and frequency analysis (on the right) in the two main groups: patients affected by neurodegenerative diseases (Deg), and patients not affected by degenerative diseases (Non-Deg + Con groups). The bar plot shows the number of patients with high and low AQP4 levels distributed in the two main groups (light for Deg and dark for Non-Deg + Con)

When the patients were categorised into three groups—with a diagnosis of neurodegenerative disease, with cognitive deficits but without a diagnosis of neurodegenerative disease, and controls—the AQP4 levels were significantly higher in the neurodegenerative group with respect to controls (Kruskal–Wallis test: *p* = 0.019) and the same result was obtained when only two groups were considered, i.e., the neurodegenerative (mean = 337.9 ± 24.9 pg/ml) versus not neurodegenerative subjects (mean = 243.1 ± 15.3 pg/ml). Mann–Whitney test between these two groups was significant, with a *p* = 0.010 (Figs. 1 and 2).

The patients were later organised into a dichotomised classification depending on the AQP4 concentrations compared to the median: AQP4-High and AQP4-Low groups were defined, and there was a significant association between neurodegenerative patients and the AQP4-High group (AQP4-High: 42 patients with neurodegenerative disease and 21 subjects without neurodegenerative disease; AQP4-Low: 28 patients with neurodegenerative disease and 33 subjects without neurodegenerative disease; chi-squared test: *χ*^2^ = 5.44, *p* = 0.020) (Fig. 2).

**Fig. 3:**
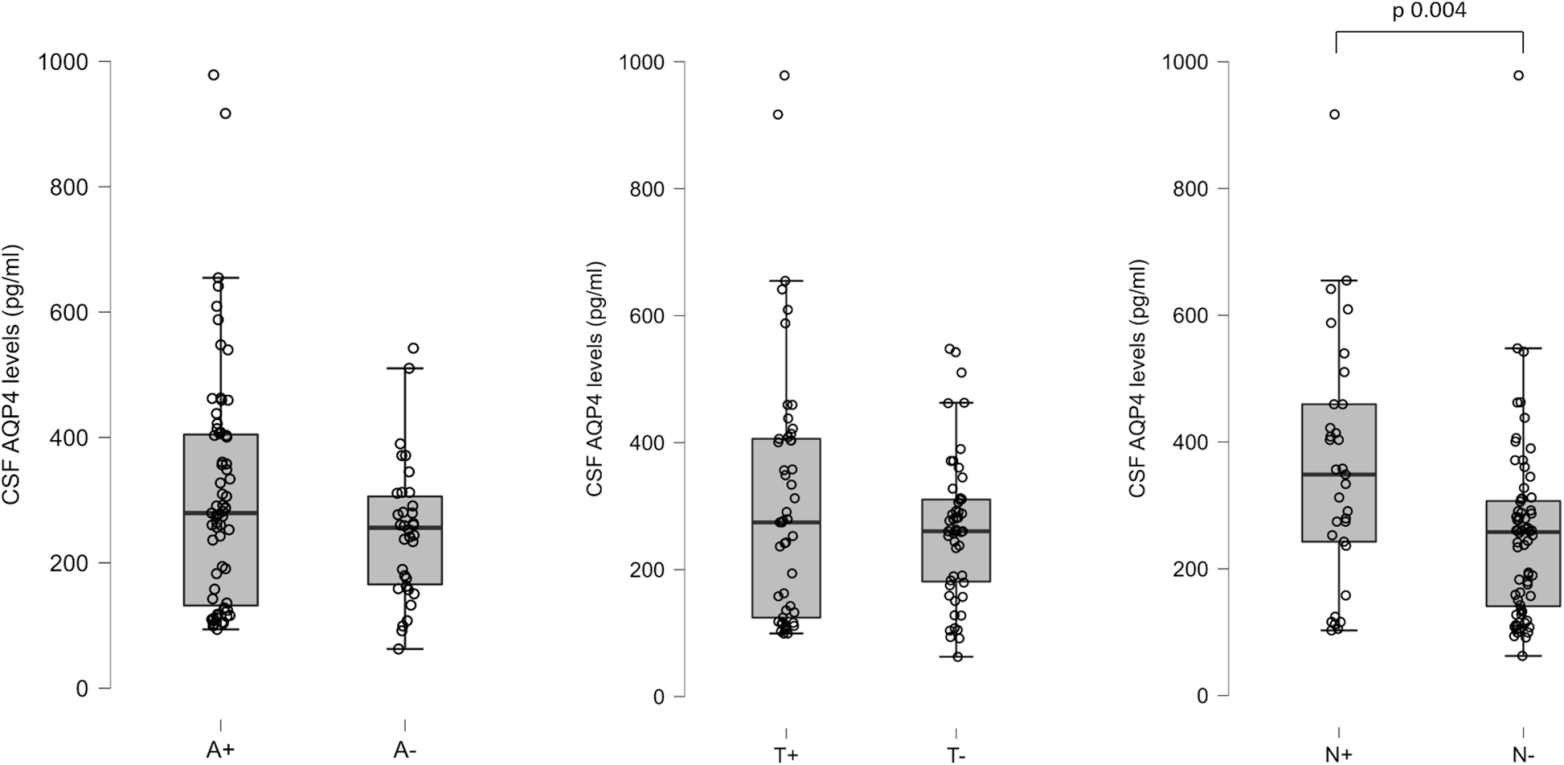
Box plots representing cerebrospinal fluid (CSF) aquaporin 4 (AQP4) levels in the three parameters of ATN classification: A + / − (according to β amyloid CSF levels) on the left, T + / − (according to P-Tau CSF levels) in the middle, and N + / − (according to total Tau levels) on the right. The only significant difference is in the levels of AQP4 in N^+^ compared to N^−^

With respect to the other CSF proteins analysed in 101 of these patients using CLEIA method, considering the differences in AQP4 concentrations with respect to the ATN classification criteria, AQP4 levels were significantly higher in the N^+^ group, which is defined by the total Tau concentration in CSF (Mann–Whitney test: A + vs A − *p* = 0.202, T + vs T − *p* = 0.622, N + vs N − *p* = 0.004) (Fig. 3).

At visual inspection, the distribution of CSF AQP4 concentrations was positively skewed; hence, for correlation and regression analysis, data were log-transformed (Log_10_); however the distribution was still non-normal; hence, non-parametric testing was preferred.

A significant positive correlation was observed between Log_10_ AQP4 levels and total Tau concentrations (Spearman’s correlation: rho = 0.186, *p* = 0.031), but no other significant correlations were observed, neither positive nor negative (Spearman’s correlation test: Log_10_ AQP4 versus Aβ1–42 rho =  − 0.158, *p* = 0.057; Log_10_ AQP4 versus Aβ1–40 rho =  − 0.034, *p* = 0.373; Log_10_ AQP4 versus phosphorylated Tau rho = 0.109, *p* = 0.140).

The same evidence was demonstrated with chi-squared association test, when comparing the ATN groups with the AQP4-High and AQP4-Low group, defined as previously mentioned (AQP4-High: 23 N + , 27 N − ; AQP4-Low: 10 N + , 41 N − ; chi-squared test: *χ*^2^ = 7.99, *p* = 0.005).

**Fig. 4:**
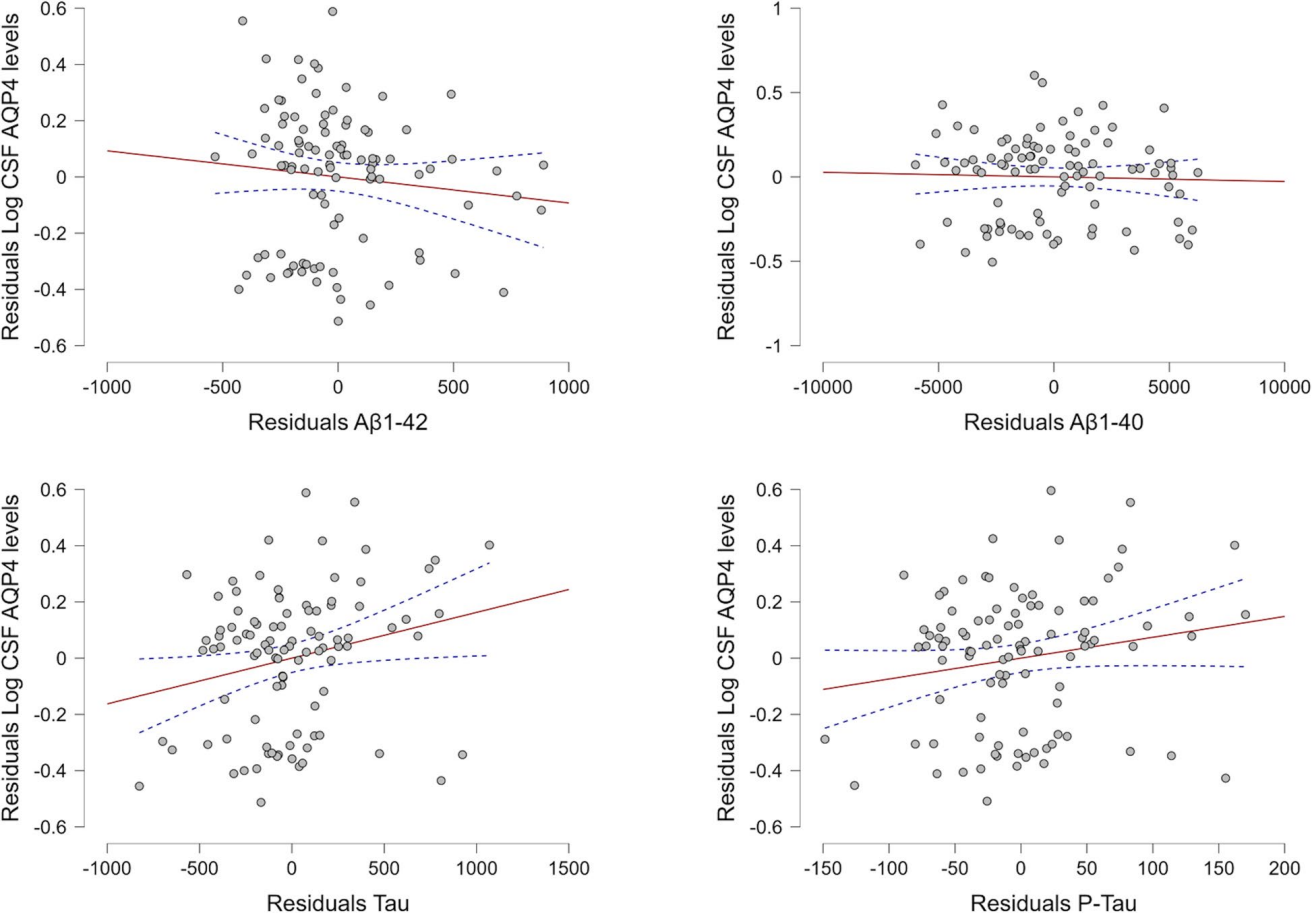
Multiparametric linear regression comparing Log_10_ cerebrospinal fluid (CSF) aquaporin 4 (AQP4) levels (*y-*axis) with β-amyloid 1–42 (first line, left), β-amyloid 1–40 (first line, right), total tau (second line, left), and phosphorylated tau (second line, right)

Multiparametric linear regressions were used to assess the correlation between CSF biomarkers and Log_10_ AQP4 levels of 101 patients whose levels of Aβ, tau, and P-tau were available with the CLEIA method, using demographic parameters as covariates (educational stage, age, sex, disease duration, and MMSE). Log_10_ AQP4 levels were positively correlated with total tau (Log_10_ AQP4 and Tau *p* = 0.026). No significant correlation was obtained when considering Log_10_ AQP4 and Aβ protein in any of its subunits (Log_10_ AQP4 and Aβ1–42 *p* = 0.308; Log_10_ AQP4 and Aβ1–40 *p* = 0.760; Log_10_ AQP4 and P-Tau *p* = 0.088) (Fig. 4).

The Kaplan–Meier estimators showed no significant differences in the rate of conversion to dementia, neither considering the AQP4-High versus AQP4-Low group, nor combining the AQP4 groups with the N parameter of ATN classification.

## Discussion

In this retrospective study, we showed that CSF AQP4 levels are significantly higher in AD patients compared to control subjects (Fig. 1). This finding was confirmed when the population was grouped according to the presence or absence of a diagnosis of neurodegenerative disease, specifically AD and FTD, two of the most common types of dementia [18–20]. This evidence may suggest a pathological change of the glymphatic system, and a consequent rise in the concentration of AQP4 in CSF, since it is crucial to its function [4, 21]. Beyond our results, very little is known about the levels of this channel protein in the CSF, as few studies focused on this topic. Bergstrom et al. [22] analysed a total of 216 different proteins in AD patients’ CSF compared to MCI patients and controls, and one of the proteins that displayed higher levels was AQP4. On the contrary, our previous study [23] found a reduction of AQP4 in CSF from AD patients compared to controls. This discrepancy could be due to the small sample size enrolled for that study (11 AD patients and 9 controls) and/or from the different methodological procedures employed. Although the technology was the same, a different ELISA kit was used that showed technical characteristics more adaptable to the analysis of molecules in the CSF.

However, the hypothesis of higher concentrations of AQP4 in CSF of people suffering from neurodegenerative disease seems more conceivable. A recent study, published in October 2021, considered an overall population of 179 individuals consisting of 81 idiopathic normal pressure hydrocephalus (iNPH), 41 AD, and 32 non-AD dementia [24]. The study argued that in these last two groups, the levels of AQP4 concentrations were undetectable in the CSF, with a mean (± standard deviation) of 0.009 ± 0.01 ng/mL for the AD patients, and 0.007 ± 0.007 ng/mL for not-AD patients with dementia, but also for the other clinical conditions examined similar negative results were obtained. This evidence prompted the authors to suggest that AQP4 is undetectable in CSF. The poor reproducibility intra- and inter-laboratory still represents a concrete limitation to be considered in a future diagnostic setting as well as the lack of a standardised methodology. These major limitations do not allow us to draw definitive conclusions on the optimal detection range of the AQP4 in CSF.

For what concerns the causes behind this evidence, not enough is known, so only assumptions can be made. One possible explanation is that reactive gliosis, a common feature in glymphatic system degeneration [6, 25], might directly induce an over-expression of AQP4 in astrocytes, which is demonstrated by an increase in its levels in the CSF. An alternative theory contemplates neurodegeneration as the cause of loss of protein selectivity on the astrocytic plasma membrane, which slows down the glymphatic flow [10, 26]. To restore it, an over-expression of channel proteins such as AQP4 would be needed, in a positive feedback mechanism.

Both these interpretations are reinforced by the association between AQP4 levels and total tau concentration found in this study, which is the very indicator for neurodegeneration in the ATN classification [16, 27, 28]. It is therefore debatable whether it is the neurodegeneration that causes a glymphatic dysfunction or vice versa, but this correlation may suggest that the two mechanisms act synergistically in a feed-forward fashion, and this is proven in this study both with clinical and molecular evidence.

No significance was found when comparing AQP4 and Aβ. Currently, the reasons behind this evidence are not known and probably involve a pathogenetic link between the two molecules. Another possible explanation may lie in the fact that patients suffering from FTD, who show hallmarks of neurodegeneration, do not necessarily have amyloid burden as well [29], so in some of these patients, high concentrations of AQP4 are not accompanied by altered levels of Aβ. It is interesting to point out that the same lack of correlation was found in the Bergström et al. study [22]. The relationship between AQP4 and Aβ was analysed in a study published in March 2021 [30], which focused on the circulating levels of AQP4 and the cerebral amyloid angiopathy-associated intracerebral haemorrhage (CAA-ICH). Despite being not able to demonstrate any significant difference between CAA-ICH patients and controls, this study showed a very interesting association between serum AQP4 levels and the load of cerebral haemorrhages in the CAA cohort; AQP4 levels were lower in the serum of patients suffering cognitive impairment, implying a very important link between Aβ clearance and AQP4 expression. The difference in blood and CSF concentrations is still to be understood, but this does not necessarily mean that the two results are incoherent with each other.

The Kaplan–Meier indicators, instead, proved no significant difference in predicting the rate of conversion to dementia. This may be the consequence of setting the median value, an arbitrary cut-off, as a discriminant for the AQP4 groups, instead of a real pathological threshold, which has not yet been identified.

Our findings are consistent with previous results concerning the expression of AQP4 in postmortem frontal cortex of cognitively healthy and histopathologically confirmed individuals with AD, in particular increasing AQP4 expression and loss of perivascular astrocytic endfeet localisation of AQP4 are associated to AD pathology [10].

Nevertheless, it is correct to underline that, although it is widely acknowledged that CSF exchanges with the brain interstitial fluid through the perivascular spaces [31], the existence of the glymphatic system is still strongly debated [32] and some crucial elements such as the parenchymal convective flow are likely to be false [33]. From this point of view, our data need to be reinterpreted, in particular the increased values of CSF AQP4 could be explained simply by reactive gliosis, in particular astrogliosis which is the enhancement of astrocytes expression with concomitant changes in its morphology [34]. Astrogliosis might directly induce an over-expression of AQP4 in the astrocytes and, consequently, an increase in its levels in CSF.

### Limitations and perspectives

The limitations of this study are the rather small sample size for the statistical analysis, although considering that we studied CSF, numbers seem reasonable. In particular, the small number of control subjects did not allow to identify an effective AQP4 cut-off value; therefore, we limited to use AQP4 median value to artificially stratify the population into subjects with high and with low levels of AQP4. A study with larger groups of patients and controls is needed to confirm these preliminary data and to identify an AQP4 cut-off value.

In addition, the choice of a case–control study to investigate a potential biomarker, undoubtedly not as effective as a prospective cohort study would be, could explain the lack of significance in the Kaplan–Meier indicators. Furthermore, despite being two of the most common causes of neurodegeneration, AD and FTD are not the only diseases with that pathological feature [35, 36]. Therefore, it is possible that they have a mechanism in common which is not shared by other neurodegenerative diseases, despite the strong association between AQP4 and tau protein suggesting otherwise.

This study has several positive aspects as well: firstly, it is one of the first analyses that tries to identify an easily accessible biomarker to characterise the newly described glymphatic system, as lumbar puncture is already being used in the diagnostic workup for people with suspected dementia [37]. Furthermore, it compared the results with other parameters widely validated, such as proteins currently used to define AD and other neurodegenerative diseases [12, 38], which contributed to the reliability of the results. Additionally, it is monocentric and used a very innovative technology for measurement, such as CLEIA [39].

Overall, these data show that AQP4 is in fact a very good potential biomarker for neurodegeneration and possibly, given its fundamental role in the glymphatic pathway [21], for the functionality of the whole system as well. This could be extremely useful in the future, as the glymphatic theory is an interesting and likely way to explain the pathogenesis of dementia and neurodegenerative disease [4], but a way to assess it in clinical practice does not exist yet.

Another interesting perspective is that using AQP4 as a marker of glymphatic functionality could help identify those common neurological diseases in which the system is compromised: for example, this study showed that, compared to controls, patients suffering from MCI or psychiatric disorders displayed a slightly, yet not significantly, increased levels of AQP4 (Fig. 1). Studies that consider larger samples focused solely on these subjects might prove a more evident difference.

Future perspectives also include the description of normal values for this protein, since there are very few studies which focused on this task [22–24], and, consequently, the definition of a pathological threshold, in order to consider AQP4 the same as other proteins, such as Aβ, tau, and P-tau, which are currently utilised. Another interesting potentiality is employing AQP4 levels for prospective, cohort studies. This may prove that not only is this channel protein possibly an early marker of disease, but it might also help identify whether it is the compromised glymphatic that causes neurodegeneration or vice versa, shedding light on one of the most controversial, yet deeply important mechanisms for neurodegeneration, especially in such common diseases like AD and FTD.

## Conclusions

This study proves that a fundamental protein for glymphatic system function and CSF and ISF flow, AQP4, is increased in CSF of patients suffering from different types of dementia compared to healthy controls, and strongly correlates with a marker for established neurodegeneration such as tau. This piece of evidence leads to a hypothesis that AQP4 could represent a fascinating potential biomarker for future studies on dementia as well as glymphatic function, and analysing this protein and its level in body fluids may help explain the pathogenesis of these diseases and hopefully provide a new tool for both diagnosis and prognosis in clinical practice.

## Data Availability

The datasets used in this study are available from the corresponding author upon reasonable request.
